# Modern Assessments of Intelligence Must Be Fair and Equitable

**DOI:** 10.3390/jintelligence11060126

**Published:** 2023-06-20

**Authors:** LaTasha R. Holden, Gabriel J. Tanenbaum

**Affiliations:** 1 Department of Psychology, University of Illinois Urbana-Champaign, Champaign, IL 61820, USA; 2 Beckman Institute, University of Illinois Urbana-Champaign, Urbana, IL 61801, USA

**Keywords:** intelligence, non-*g*, process overlap theory, cognitive assessment, non-discriminatory assessment, equity and fairness, diversity and inclusion

## Abstract

Historically, assessments of human intelligence have been virtually synonymous with practices that contributed to forms of inequality and injustice. As such, modern considerations for assessing human intelligence must focus on equity and fairness. First, we highlight the array of diversity, equity, and inclusion concerns in assessment practices and discuss strategies for addressing them. Next, we define a modern, non-*g*, emergent view of intelligence using the process overlap theory and argue for its use in improving equitable practices. We then review the empirical evidence, focusing on sub-measures of *g* to highlight the utility of non-*g*, emergent models in promoting equity and fairness. We conclude with suggestions for researchers and practitioners.

## 1. Introduction

Achievement gaps in cognitive assessments and standardized tests have been documented for decades with Black and Hispanic students performing worse compared to White and Asian students (Hunter and Bartee 2003; Ladson-Billings 2006; Berlak 2005; Lee 2002). Additionally, gaps in achievement between immigrants and non-immigrants, and native and non-native speakers have also been found (Marks 2005; Levels and Dronkers 2008; Borgonovi and Ferrara 2020). Importantly, the population of the United States is becoming increasingly diverse and, by the year 2050, racial minorities including Black, Hispanic, and Asian people are projected to make up a majority, with Black and Hispanic groups increasing most significantly (Vespa et al. 2018). In addition to these demographic shifts, diagnoses for attention-deficit hyperactivity disorder (ADHD), autism spectrum disorder (ASD), and intellectual disabilities all increased in children from 2009 to 2017 (Zablotsky et al. 2019). This suggests that current assessment practices are unlikely to be fairly or equitably assessing current examinees. These shifting demographics along with the persistent achievement gaps point to a need for a greater focus on making intelligence assessment more inclusive and equitable to better serve the needs of all students.

When examining forms of cultural and neurodiversity, piles of empirical evidence suggest that cognitive functions and processes are oriented and developed in context; thus the deployment of cognitive skills and strategies will occur in different ways (Ortiz and Oganes 2022; Wang 2021; Gutchess and Rajaram 2022; see also Washington et al. 2018; Prather 2021; Thomas et al. 2023). We argue that when considering equity and fairness this must be taken into account in cognitive assessment research and practice. To do this, we argue that basic science and research from cognitive psychology should be applied to cognitive assessment research and practice. Further, we argue that a non-*g* emergent perspective of intelligence with the process overlap theory (POT) can be applied to help us determine how to conduct scientific research and assessment design and practice in more fair and equitable ways. We will build our arguments for this, starting with a review of intelligence theories and assessments. We will then discuss how to make intelligence assessment fairer for diverse groups. We will conclude with suggestions for researchers and practitioners.

## 2. Intelligence Defined as General Ability from the Positive Manifold

One of the pioneers in intelligence research, Charles Spearman (1904) claimed to have objectively measured and defined intelligence. Using data from children’s school grades, Spearman observed that performance in one cognitive ability task correlated positively with performance in other cognitive ability tasks—a phenomenon called the positive manifold. The positive manifold has been replicated many times across different contexts, cultures, and populations (Pluck and Cerone 2021; see also Jensen 1998). Because of this, there is possibly something universal about the positive manifold making it a special aspect of human cognition.

Based on this work, Spearman (1904) coined the notion of psychometric *g* or the general factor of intelligence, which was believed to be the single, hierarchical factor responsible for performance on cognitive tasks. This led to views that psychometric *g* is unitary and, ultimately, the innate cause for the scores and differences between them that we observe in cognitive tasks from one person to the next. This *g* factor was derived mathematically from the variance shared between different cognitive tests (Spearman 1927). As such, the *g* factor was thought to reflect the fact that a person’s performance in one cognitive task is usually similar in other tasks. In this model of psychometric *g*, the latent variables are reflected by multiple measurements on a certain cognitive task. This made the *g* theory of intelligence a reflective model, where the *g* factor is at the top and is reflected in lower-level processes. In contrast, formative models have an opposite relationship between scores in cognitive tasks and latent variables—designating *g* as emergent based on the positive manifold rather than as their cause (Kovacs and Conway 2016).

## 3. Spearman and Thomson

Another theory of intelligence that developed as a response to Spearman’s *g* model, was Godfrey Thomson’s (1916) bonds model of intelligence. The bonds model of intelligence stated that cognitive tests sampled from a variety of elements (or bonds). The idea is that the purpose of assessing intelligence should be to sample from various sorts of elements that make up cognitive ability. This model proposes that different cognitive processes (seemingly domain general and specific) are sampled and engaged in different tests and as such the positive manifold is thought to be a function of shared bonds. From this model it was suggested that each bond had an equal probability of being sampled by different items in a cognitive test. In turn, Thomson argued that the general factor *g* was not needed to explain the positive manifold. Thomson’s model did not receive much attention at the time because it was difficult to understand, but was later revisited (see Bartholomew et al. 2009; Van Der Maas et al. 2006). Since Spearman and Thomson, there have been many other proposed theories and tests of intelligence; next, we will discuss some of the more prominent ones.

## 4. Models and Measures since Spearman

The Binet–Simon scale was developed in 1905 and was the first formal measure of intelligence, with its creator, Alfred Binet widely considered as the father of intelligence tests (Wasserman 2018). The Binet–Simon scale contained 30 items, combining a variety of cognitive tasks to provide a measure of the intellectual capacity of a child. Although the original Binet–Simon scale did not include an intelligence quotient (IQ), the 1908 revision allowed for the estimation of a child’s mental level based on a 75% pass rate on each subtest (Binet and Simon [1908] 1916). This was based on the normative performance of only 200 children and was later referred to as a “mental age level” in the United States, although the original sample was from France. This procedure of using a standardization sample became the conventional practice for developing intelligence tests moving forward and has since faced criticism (Graves et al. 2021; Graves 2022). Binet’s original scale was adapted and translated many times and, by 1939, there were 77 versions. The most popular was the Stanford–Binet, the most widely used intelligence test in the U.S. in the 1920s and 1930s. Based on the problematic views of eugenics (see Galton 1869), intelligence testing was used in harmful and unjust ways. The Stanford–Binet test was used to separate students with learning disabilities and later as justification for forced sterilization of people with learning disabilities and racial minorities by the U.S. Supreme Court (Black 2004). This occurred even though its creator, Alfred Binet, was one of the few intelligence scientists who rejected eugenics (Russell 2009).

Later, Cattell–Horn–Carroll (CHC) theory was developed, combining the work of John Carroll’s three-stratum theory with the fluid and crystallized intelligence (Gf-Gc) theory from Raymond Cattell who further developed the theory with John Horn (Wasserman 2018; see also Horn 1965; Cattell 1941, 1963). The CHC theory states that cognitive abilities can be classified into three strata ranging from narrowest to broadest: stratum I consists of many specific factors, stratum II consists of eight broad cognitive abilities that contribute to *g*, and stratum III consists solely of Spearman’s *g* (Schneider and McGrew 2018). The CHC has a hierarchical structure for the dimensions of abilities and is thought of as a reflective model with the *g* factor at the top as the common cause of abilities in the lower strata.

The Woodcock–Johnson (WJ) tests of cognitive abilities, currently in the fourth edition, are based on the CHC theory of intelligence. The WJ is the only cognitive assessment measure that has scores associated with all seven broad ability domains found in the CHC (Schneider and McGrew 2018). The assessment contains 10 standard battery subtests which combined produce cluster scores for crystallized ability (Gc), fluid reasoning (Gf), and short-term working memory (Gsm/wm), in addition to a general intellectual ability (GIA) composite score which is thought to be a proxy for *g* (McGill 2023).

The Cognitive Assessment System (CAS) is another tool for assessing cognitive abilities, first published by Naglieri and Das (1997) and now in its second edition (CAS2, Naglieri et al. 2014). The CAS was developed based on the planning, attention, simultaneous, and successive (PASS) neurocognitive theory of intelligence. The CAS was designed with the view that a theory of intelligence should be the basis for a test of intelligence and such tests should measure “basic neurocognitive processes defined by the intellectual demands of the test, not the content of the questions” (Naglieri and Otero 2018). Scores from the CAS can be used to predict achievement test performance; produce unique profiles for children with learning disabilities; and have other applications, such as planning instruction and interventions for students. Though the CAS has these advantages, it is not as popular or widely used as other tests.

Two other prominent intelligence tests used today are the Wechsler Intelligence Scale for Children, fifth edition (WISC-V) and the Kaufman Assessment Battery for Children, second edition (KABC-II). The WISC-V aligns its scales with the factor-analytic work of Carroll–Horn (Kamphaus et al. 2018). The KABC-II is also based on the CHC intelligence theory (Schneider and McGrew 2018). Though CHC is one of the most prominent theories of intelligence today, there have been previous attempts to model intelligence in different ways.

## 5. Other Models of Intelligence

Other researchers have worked to develop more innovative and inclusive models of intelligence. One such model, Gardner’s (1983) theory of multiple intelligences differs from the traditional idea of intelligence by defining it as “a biopsychological potential to process information that can be activated in a cultural setting to solve problems or create products that are of value in a culture” (Chen and Gardner 2018; Gardner 1999). Due to this definition of intelligence as a potential, this theory suggests that intelligence is emergent and responsive rather than innate and fixed. Although this theory is unique and interesting, it is difficult to test in many cases, which has made it hard to support with empirical evidence (Visser et al. 2006; Davis et al. 2011).

Another unique intelligence theory is the Sternberg triarchic theory of successful intelligence, first published in 1985 and consisting of three parts: componential, experiential, and practical (Sternberg 2018). Sternberg defines intelligence as “(a) mental activity directed toward purposive adaptation to, selection and shaping of real-world environments relevant to one’s life” (Sternberg 1985). In triarchic theory, psychometricians and psychologists seek to understand the processes underlying mental states and intelligent thought. This theory, like Gardner’s, has not been translated to clinical intelligence tests due to difficulties in measurement. However, it should be noted that triarchic theory provides a culturally inclusive approach to thinking about intelligence as it directly acknowledges that adaptive thought or behavior may vary between cultures and different contexts shape thoughts and behaviors differently.

More inclusive theoretical perspectives of intelligence are important, and we argue that although culture and context have been acknowledged (e.g., see Sternberg and Grigorenko 2004) they remain largely underappreciated in assessment research and practice. Next, we review the history of the design and implementation of assessment practices to better understand their limitations for making progress toward equity and fairness. We will then summarize the limitations of these models and their practices to motivate the current definition and theory of intelligence used in this paper.

## 6. Diversity, Equity, and Inclusion Concerns in Cognitive Assessment

For whom were cognitive assessments designed historically? It is known that Spearman developed his model and theory based on students in the U.K., some of whom attended private preparatory schools. These students were mostly White and likely of a certain social class in or around the late 1800s. Hence, Spearman’s model and theory were developed from students of this time and that is the context from which his data came. A lot has changed since then, yet this model of intelligence has been applied to the U.S. context in some form or another over many generations into modern times.

In 2014, the U.S. hit a milestone where students in school were no longer majority White and became majority–minority (Maxwell 2014). Forms of neurodiversity have been on the rise as well—those with learning and developmental differences have shown recent population increases, with 13% of public-school students served under the Individuals with Disabilities Education Act in the 2009–2010 school year compared to 15% in the 2020–2021 school year (Irwin et al. 2022). These data demonstrate the increasing forms of cultural and neurodiversity in the U.S. and point to the need to find ways to fairly assess a variety of learners.

Further, students who have multiple marginalized identities, including Black and Hispanic students, are found to be overrepresented in special education (Cortiella and Horowitz 2014). This overrepresentation has been linked with biases in referral processes, aspects of assessment and placement, teachers’ judgment of student abilities based on race, and unfair testing that may be biased towards students of certain backgrounds (Arnold and Lassmann 2003; Cooc 2017). These findings raise questions as to whether individuals from these identity groups are being adequately and fairly served through assessment practices, let alone fairly included and represented in assessment research.

Today, the Binet and Wechsler scales are still the predominant intelligence tests used in American schools despite their many problems including inappropriately and disproportionately placing low-income and minority students in special education, which leads to fewer and less enriching educational opportunities (Sireci and Randall 2021; Slavin 1987; Darling-Hammond 1986). There have also been multiple court cases regarding the use of IQ tests in schools (see Hobson v. Hansen 1967; Diana v. State Board of Education 1970; Larry P. v. Riles 1979). Specifically, the Larry P. case led to the outlawing of intelligence testing of African-American students in California, which remains in effect to the present day (Sireci and Randall 2021). These tests had consequences outside of education as well, including their use to restrict access for Black jobseekers (see Griggs v. Duke Power Co. 1971; Bond 1987). These are just a few examples of the many injustices that have been legitimized using intelligence tests.

In light of many concerns regarding injustice, several groups have argued against the use of intelligence tests. The Association of Black Psychologists supports parents who have refused to allow children and themselves to be subjected to intelligence tests (Council of National Psychological Associations for the Advancement of Ethnic Minority Interests CNPAAEMI 2016). In fact, in 1969 the Association of Black Psychologists called for a moratorium on the administration of ability tests to all Black students due to inherent racial biases (Sireci and Randall 2021). It is crucial to acknowledge these concerns about inequity and injustice given the dark history of intelligence assessment, its applications, and the effect this has had on many lives. Considering this and the growing forms of diverse needs, we argue that reform of assessment design and practice is urgent, and that reform efforts must be directed toward goals of equity and fairness (see also Holden and Hart 2021). Thus, rather than calling for the elimination of cognitive ability testing (see McGrew 2023; McGrew et al. 2023 on the death of cognitive ability tests being premature), researchers and practitioners must find ways to make them better suited for students of all backgrounds.

Recognizing that Spearman’s theory and model of intelligence were developed from a particular sample, within a certain cultural group and context, it is also clear that it has not been expanded, applied, or translated into practice in the most equitable of ways. Therefore, when thinking about for whom cognitive assessments can be designed today and where to start, we argue that we should begin with moving past outdated and unfair methods and practices. However, how do we define equity and fairness in cognitive assessment? We will consider this question and discuss how equity and fairness have been examined in current assessment practices.

### Thinking about Equity and Fairness in Assessment Research and Practice

The concept of fairness involves issues related to validity, which includes reflection on what is fair in both how tests are developed and used (AERA et al. 2014). For example, one important question around validity and test scores is whether there is adequate representation of subgroups in the normalizing samples used in the test development process. This calls into question construct validity and whether the interpretation and use of test scores from subgroups that may not be sufficiently represented in normalizing samples, is truly fair. Work is still being carried out to uncover whether applying certain forms of intervention are possible and beneficial for racialized students based on them being under-represented in research and assessment development practices (see Lindo 2006; Proctor et al. 2012).

Fairness in assessment research and practice can also be considered based on measurement, legality, and/or philosophical issues (Worrell 2016). Worrell (2016) highlights four main components for fairness and validity based on the AERA et al. (2014) *Standards for Educational and Psychological Testing*: (1) fair treatment during testing, (2) fairness in terms of lack of measurement bias in scores, (3) fairness in accessing the construct, and (4) validity of individual score interpretations for intended uses. Fair treatment during testing means that all examinees can show what they know on a test, with standardization of test administration and scoring. Fairness in terms of lack of measurement bias can be achieved by examining scores for differential item functioning (DIF) and differential prediction, different estimates in precision, factor structure, and looking at differences in the meaning of construct. Fairness in accessing the construct means considering questions such as: does each examinee have the same access in terms of opportunity to show their true standing on the construct, and has construct irrelevant variance (CIV) been minimized? This point is especially lacking in current practice and needs more attention. The CIV is a type of systematic error introduced in an assessment by variables that are not related to the construct measured (Downing and Haladyna 2004). For example, item bias may be one source of CIV that is of particular interest when investigating equity and fairness.

The AERA et al. (2014) also add two major concepts that are useful for minimizing bias (and thus increasing fairness) which are accessibility and universal design. Universal design emphasizes the need for developing tests that maximize usability for all test takers, regardless of characteristics such as gender, age, language background, culture, socioeconomic status, or disability. Principles of universal design include precisely defining constructs, avoiding (when possible) item characteristics and formats or test characteristics that may bias scores for individual subgroups, and minimizing challenges by taking into account test characteristics that may impede access to the construct for certain test takers. However, universal design for more typical forms of assessment (such as standardized tests) still often create disproportionality with racialized and marginalized students being overly identified for special education. Therefore, we must be thoughtful about what kinds of tests to use and when they are used (Worrell 2016). Based on the many concerning and problematic forms of bias historically, we next examine what has been done to make commonly used assessments fair and equitable in current practices.

## 7. Examining Equity and Fairness in Current Assessment Practices

In a 2018 survey, school psychologists were asked to indicate which assessments they believed were culturally appropriate and fair for Black students, the most frequently mentioned were: The Kaufman Assessment Battery for Children, 2nd edition (KABC-II), Differential Abilities Scales (DAS), and the Cognitive Assessment System (CAS). Interestingly, none of these were the most frequently used assessment, which was the Wechsler Intelligence Scale for Children 5th edition (WISC-V) (Aston and Brown 2021). This is a problem because the WISC-V has been found to contain several verbally loaded items which could have downstream consequences including disparate forms of educational placement (Grissom and Redding 2016; Arnold and Lassmann 2003). Many of these test items contain cultural- and context-specific language to which Black students in particular may have less exposure (Aston and Brown 2021).

In an earlier work, Willson et al. (1989) examined race and gender effects on the K-ABC. They found that several items were biased, 23 against Black examinees and 13 against Whites. It was concluded that “although the effects of these biases were statistically significant, the biased items were essentially counterbalanced, eliminating the effect such items would have on race or sex differences in mean scores”. The use of counterbalancing items appears insufficient for thoroughly addressing bias in intelligence tests especially given the sophistication of assessment design and development methods and statistical techniques available today. Differential item functioning and differential test functioning analysis are two such methods that may be employed to test for bias and fairness on assessments (Haughbrook 2020; AERA et al. 2014).

In a more recent study, Scheiber (2016) looked at White, Black, and Hispanic students’ performances in the KABC-II and the Kaufman Test of Educational Achievement 2nd edition (K-TEA II), the tests were found to be unbiased for all three groups through factorial invariance of the factor structure, which is based on seven broad abilities from the CHC theory of intelligence. The matched samples used in the KABC-II test development process found a small between race score difference of 5.0 points which is around half that of other tests (Lichtenberger et al. 2009). Another study by Dale et al. (2011) found that African-American and White children performed similarly on the KABC-II. Although the between group performance gap was not eliminated, this provides further support for the Kaufman tests in order to provide a fairer test of cognitive abilities for minorities.

However, when examining racial/ethnic differences between different tests of ability, the smallest between-race differences were found for the CAS2 (Naglieri and Otero 2018). This was found when looking at the scores in the standardization sample for the CAS2 (after controlling for other demographic variables), with only a 4.5 point gap between African-American and non-African American children (Naglieri et al. 2014). This is a marked improvement compared to the WISC, Stanford–Binet, and WJ, with between-race score differences for the CAS2 about half of those found for the other tests. The CAS2 was able to achieve these improvements by using a cognitive processing approach for measuring ability compared to other tests that require higher demands on verbal and quantitative knowledge similar to standardized achievement test knowledge requirements (see Naglieri and Bornstein 2003). This work suggested that focusing specifically on cognitive processes and reducing the demands for forms of cultural or context-specific knowledge helps make this assessment more accessible and fairer for students.

The Council of National Psychological Associations for the Advancement of Ethnic Minority Interests (CNPAAEMI) has also called the CAS and KABC-II relatively more culturally fair than other intelligence tests because they have both shifted towards neuropsychologically based approaches that are less reliant on academic tasks. Both the CAS and KABC-II have been found to still correlate well with academic achievement while also producing significantly smaller gaps between Black and White children’s scores (Naglieri and Bornstein 2003). Further, the KABC-II was designed with inclusivity in mind and aimed to minimize the cultural gap between White and Minority children (Lichtenberger and Kaufman 2010). The ways in which this was accomplished included: elimination of knowledge-based subtests from global score indices, reduced emphasis on language and crystallized abilities for measuring overall cognitive ability, subtests designed based on research with fewer cultural differences (e.g., face recognition and gestalt closure), reduced verbal load for both examiner and examinee, and inclusion of teaching items where examiners are encouraged to modify wording, use gestures, and explain introductory terms. Taken together, this shows that there are known and proven methods for arriving at intelligence assessments that are more inclusive, culturally sensitive, and fair across diverse groups of students.

### 7.1. Limitations and Challenges

Although there are some positive findings related to equity and fairness in these commonly used assessments, there are also many noteworthy limitations. A 2021 study by Graves et al. on whether the WISC-V is fair for Black children found evidence that did not support measurement invariance in cognitive abilities across urban Black and White youth. They also mentioned that a major problem with the WISC-V is its over-reliance on standardization samples and that previous versions had had more independent studies carried out to investigate the validity/stability of the scales. The standardization sample for the WISC-V includes racial proportions based on the United States population, which includes 13% African-American children (Graves et al. 2021). Even though that 13% is representative of the whole country, this does not provide sufficient representation for more diverse sub-populations, such as urban schools that have larger Black populations. Graves et al. focused on this in their study and had 55% Black/African-American subjects. This allowed them to better study the WISC-V for Black students.

A study by Woods et al. (2021) found that the CHC-based model of the WJ IV does not provide an acceptable prediction of reading achievement in the nine to thirteen-year-old age group, including for Black, White, and Hispanic children. This is important for equity considerations with the CHC theory because a lack of predictive ability of the assessment across different groups calls into question the utility of those measures. Haughbrook (2020) used differential item functioning analysis to look at racial bias of the WJ III (specifically the Picture Vocabulary scale) and also found a significant bias against Black students. Another study that looked at the WJ IV performed factor analysis on the test battery (Dombrowski et al. 2017). The results of this study found a factor structure that only partially aligned with the theoretical structure in the WJ technical manual and raised concerns about the WJ IV alignment with CHC theory itself.

A noteworthy feature of the WJ is the fact that it is referred to as “tests of cognitive abilities” which implies the importance of assessing all broad abilities and lends itself to a more formative notion of intelligence compared to other tests based on CHC that emphasize psychometric *g* such as the WISC which calls itself an “intelligence scale”. Others have called into question CHC theory itself, with problems including its application in cross-battery assessment (Glutting et al. 2003) as well as theoretical disagreements and incongruencies between the separate theories of Carroll and Cattell–Horn (Canivez and Youngstrom 2019). The Cattell–Horn approach also denies *g* which is included in CHC. Although the WJ IV was created to specifically represent CHC, the independent follow-up studies of it did not provide adequate support for the CHC model (see Dombrowski et al. 2018a, 2018b; Canivez 2017).

Looking at work on the CAS, previous studies have performed confirmatory factor analysis in order to determine what constructs it best measures. One such study by Kranzler and Keith (1999) found that data from the CAS did not fit well to the PASS model and that the best fit could be found using CHC. Another study by Keith et al. (2001) found further evidence that the CAS does not have construct validity as a measure of the PASS processes. Results from both analyses call into question the practical utility of the CAS for planning educational intervention and learning disability differential diagnoses. The results of these studies also challenge the validity of the PASS model itself as a theory on individual differences in intelligence.

### 7.2. Summary

Berlak (2001) states that it will be difficult to help close achievement gaps because current forms of cognitive assessment are steeped in a history of cultural and racial bias. As outlined above, common cognitive assessments have attempted to remediate fairness and equity problems by reducing verbal load, reducing unnecessary focus on specific forms of crystallized knowledge, and by focusing more on the cognitive processes of intelligence. We argue that these strategies should be incorporated and improved upon in future work. Further, progress toward equity cannot be achieved without more substantive reform efforts by test developers and practitioners. We argue that the appropriate reform should begin with how we define intelligence, how we study it scientifically, and how we apply it in assessment design and practice. Cognitive assessments of intelligence do not have to be used for harm and they can be designed and developed to be used in beneficial and equitable ways. We seek to provide evidence from recent scientific theory and research in order to support this goal.

Based on the substantial theoretical work and experimental evidence on cognitive processes developed from cognitive psychology research (Engle et al. 1999; Engle 2002; Kovacs and Conway 2016; Kovacs et al. 2019), it appears that this work has been largely ignored when it comes to assessment development and practices. We argue that this should not be the case and that evidence garnered from this literature should be more seriously considered in assessment development and practice in the future (see McGrew et al. 2023). We will use the process overlap theory of intelligence and adjacent empirical evidence from cognitive psychology to make our case for it informing more fair and equitable assessment research and practice.

## 8. Intelligence Defined as Emergent through Process Overlap Theory

The process overlap theory (Kovacs and Conway 2016) expanded aspects of Thomson’s model (Thomson 1916) and defined intelligence as emergent from the interaction of many cognitive processes. Kovacs and Conway note that rather than all cognitive bonds being sampled equally as Thomson believed, instead the domain-general processes are sampled more during a test than domain-specific processes and the domain-general processes overlap more with domain-specific processes than the domain-specific processes overlap with each other. Process overlap theory (POT) is a new account of *g* and as an emergent model of intelligence, it does not require the hierarchical structure suggested from reflective models. Instead, here, the *g* factor is a statistically emergent property based on the positive manifold, making *g* not required to explain the positive manifold. As such, POT can be thought of as a non-*g* model. We argue that non-*g* POT is a better model due to it being more parsimonious (see McGrew et al. 2023), being better for equity, and because it shows us where to focus when we consider forms of cognitive optimization or enhancement through intervention. Next, we will support our arguments for POT in more detail considering how subprocesses of *g* can be applied to better use intelligence for more equitable and fair assessment research and practice.

### 8.1. The Utility of Focusing on Subprocesses of g

Contrary to the view of intelligence as innate (see Galton 1869) and *g* as the common cause responsible for performance on all cognitive tasks (see Jensen 1998), POT allows us to examine individual strengths and weaknesses that emerge across a variety of subprocesses of *g*. Kovacs et al. (2019) argue that ability differences occur because the lower the ability on the central executive process, the lower the probability of solving a cognitive task correctly—regardless of the level of ability on domain-specific processes. To further complicate things, researchers in cognitive psychology argue that any assessment of cognitive ability is not process pure (Mashburn et al. 2021). This means that to some extent every assessment item taps into a variety of different cognitive processes and depending on the item, some will be more involved than others. Because of this, we should focus on which processes are most implicated for a broad range of tasks. In other words, we should prioritize the cognitive processes that appear most important for *most* cognitive tasks.

Additionally, the central executive processes (e.g., executive attention or cognitive control processes) in working memory capacity (WMC) and intelligence are thought to limit performance in a general way (Kovacs et al. 2019). In turn, making errors is more likely at lower levels of domain-general executive ability regardless of the domain-specific processes tapped by the same tasks. This means that the controlled processes are the most important to prioritize because impacting them will cause the biggest hit on cognitive performance. Recent work also emphasizes the strong relation of fluid intelligence (gf) with working memory, arguing this is underpinned by their reliance on domain-general executive attention/attentional control (Mashburn et al. 2021; Engle et al. 1999). By extension, we can better understand complex cognition from a POT perspective by focusing first on the two control mechanisms of maintenance of relevant information and disengagement with irrelevant information (see Shipstead et al. 2016).

Considering the most important executive processes, previous research emphasizes executive/attentional control, working memory, maintenance, and disengagement, which are all thought to collaborate in completing a task goal (Shipstead et al. 2016; Mashburn et al. 2021; Engle 2018, 2002; Martin et al. 2020). During task completion, working memory is heavily involved in maintenance processes whereas disengagement is thought to heavily involve fluid aspects of intelligence (Shipstead et al. 2016). Executive attention or attentional control processes are defined as being able to organize cognitive processing based on objectives (Shipstead et al. 2016). Working memory on the other hand is thought of as the workbench of the mind. Working memory has a capacity component (WMC) and enables the ease of concurrent processing allowing for storing, retrieving, manipulating, and updating of information (Baddeley and Hitch 1974; Baddeley 2001). Finally, disengagement is defined as “the act of removing information from ongoing cognition so that new information (i.e., more relevant information) can exert a greater influence” (Shipstead et al. 2016).

Another pertinent consideration is the role of domain-specific processes and their interaction with these domain-general processes in bringing to bear successful goal achievement (Kovacs and Conway 2016). It is not that domain-specific skills are not important; however, based on previous work, they are not shown to account for the same degree of predictive power as domain-general processes (Mashburn et al. 2021; see also Turner and Engle 1989; Daneman and Carpenter 1980). Likewise, Kane et al. (2004) suggested that WMC is a more general concept and argued that this is due to individual differences in domain-general executive attention. Moreover, though working memory is thought of as a domain-general construct, it can also be decomposed into modality-specific control processes. Previous research finds little overlap between the working memory of different modalities, such as verbal vs. visuospatial working memory (Shah and Miyake 1996). Instead, verbal and visuospatial working memory use different coding schemas, representational format, and also possibly different storage systems (Shah and Miyake 1996). This indicates there might be additional considerations for future research and forms of intervention and patterns of individual strengths and weaknesses in more specific cognitive abilities.

Considering this literature on working memory and general executive processes having major impacts on performance, this emphasizes the need for greater focus on understanding the role of these processes and their application in assessment practices. With a non-*g* perspective, POT reveals what should be prioritized in assessment design from these domain-general processes. In future work, we suggest that these domain-general processes be further examined in addition to considering previous forms of evidence-based practices for increasing fairness including additional focus on cognitive processing approaches, reducing the verbal load and other unnecessary loads based on cultural- and context-specific forms of acquired knowledge (Naglieri and Bornstein 2003; Lichtenberger and Kaufman 2010). Next, we will support our argument for focusing on subprocesses of *g* with additional empirical evidence considering parsimony, equity, and fairness.

### 8.2. Focusing on Subprocesses Is More Parsimonious and Better for Equity and Fairness

Due to psychometric *g* (Spearman 1904) being revealed as statistically emergent based on the positive manifold, it is not only “not real” (Kovacs and Conway 2016) but not necessary for the study or best understanding of cognitive performance. As such, we argue that a non-*g* model is better for a variety of reasons. One such benefit is that it is more parsimonious. Hierarchical *g* and non-*g* are shown to be statistically equivalent. Therefore, the hierarchical structure as often suggested in the commonly used CHC model is not necessary when just as much of the variance can be explained without it (McGrew et al. 2023). Thus, as a non-*g* model that is well supported by basic scientific evidence (Burgoyne et al. 2021, 2022; Shipstead et al. 2016; Mashburn et al. 2021; see also Martin et al. 2020; Peng and Swanson 2022), process overlap theory is a more parsimonious approach to thinking about assessment. Further, considering the utility of examining subprocesses of *g*, as outlined above, non-*g* POT is also better for working toward equity and fairness goals. Next, we discuss this in more detail with supporting evidence.

Work by Kovacs et al. (2019) argues that if domain generality of the working memory is influenced by capacity, then the relative provision of subprocesses to the WMC are not universal, indicating that these processes are not the same for different people. This is because the limits of WMC may reflect different mechanisms in different people. We believe this point is critical when considering forms of equity and fairness in assessment. If there are differences in the baseline domain-general executive processes and these impact the functions of the WMC, then more care needs to be taken in considering this in assessment development and practice. This also brings up the question of how to identify the intra-individual processes responsible for WMC, considering Kovacs et al.’s point about non-universality. Determining this might be harder than previously thought, because the mechanisms that underlie WMC vary within person and might even vary as a function of different control or executive function limits in capacity.

### 8.3. Empirical Evidence Supporting Subprocesses Based on Non-g POT

From a perspective of equity and individual differences we already know that working memory has been found to be harmed by forms of racial bias (Schmader and Johns 2003) but is also shown to be important for mental resilience during identity-threatening situations (Beilock et al. 2007; Régner et al. 2010; Holden et al. 2020). Likewise, in a study of students in an urban context, Graves et al. (2021) found evidence that fluid intelligence and working memory operate differently for Black students, suggesting possible measurement bias across racial groups. The finding of differences in such important domain-general processes in a “gold star” cognitive assessment such as the WISC makes it difficult to view scores on this scale as fair, let alone valid. This points to the need for more research focused on understanding diversity in cognitive functions and processes, potential cultural and contextual differences in these abilities at baseline, as well as how they are leveraged and deployed.

Others have also suggested that a focus on subprocesses of *g* can be beneficial for equity in the domain of high-stakes testing, particularly for personnel selection and classification-based decisions. Burgoyne et al. (2021) show that shifting the focus to domain-general subprocesses of *g* and moving away from crystallized measures (that focus on more culturally specific acquired knowledge) is better for reducing forms of adverse impact. Specifically, they show that tests that focus on attentional control and domain-general executive processes involved in maintaining focus on task-relevant information (e.g., working memory tasks) provide a more equitable approach to cognitive assessments. Moreover, additional work by Bosco et al. (2015) shows that focusing on the domain-general executive processes of attentional control and working memory was better for reducing adverse impact between racial/ethnic groups compared to using more traditional assessments that emphasize psychometric *g*. Other work by Burgoyne et al. (2022) provides more support for domain-general subprocesses for reducing the adverse impact and improving the variance accounted for in academic performance above and beyond traditional cognitive assessments in a sample of Navy trainees. This work also found that attentional control measures improved training classification accuracy in trainees and reduced subgroup differences between minority and majority members. Taken together, these studies provide empirical support for focusing on subprocesses of *g*, and specifically for those that POT would suggest are most important for optimal performance and reducing bias.

The theoretical foundation applied in cognitive assessments is also of great importance for equity and fairness concerns. Strong, theoretical underpinning allows for a clearer interpretation of test results and makes way for a more specific path for intervention support. The importance of a strong theoretical underpinning was recently demonstrated in work by Moore and Conway (2023). Their goal was to study the structure of neurocognitive abilities and their relationship with childhood behavior problems using secondary data from the Adolescent Brain Cognitive Development Study (ABCD) (2021) study. They examined the difference in results when modeling cognitive ability based on a more theoretically motivated approach with POT compared to a less theoretically motivated approach which focused on “general cognitive ability” (Thompson et al. 2019). Moore and Conway show that when researchers investigate the structure of cognitive abilities ignoring the literature on theory and basic science in cognitive psychology, this not only contributes to inappropriate hypotheses but also leads to inappropriate model fitting procedures. For example, basic science in cognitive psychology and intelligence demonstrates that cognitive abilities are positively correlated and that model fitting which assumes cognitive ability measures are orthogonal is inappropriate (as in the Thompson et al. 2019 work). Secondly, they point out the importance of understanding theories in cognitive psychology and intelligence when developing novel hypotheses around the structure of cognitive abilities. For example, Thompson et al. model the data claiming the first component in their model is general ability. This is inconsistent with prominent theories of intelligence and cognition which state that general ability or *g* is the higher order factor that is thought to reflect variability among the lower-level broad ability factors in the CHC model. On the other hand, the POT account would view *g* or this so-called general ability factor as only statistically emergent and not real.

As Moore and Conway illustrate, it is imperative that future research on the structure of cognitive abilities consider basic science and theory in cognitive psychology. Otherwise, we are doing a disservice to students whose lives could be impacted by the inferences drawn, as well as the interventions and different assessment practices developed from them. This could have real negative consequences for the future trajectory of students’ lives. From this work, Moore and Conway also highlight the limitations of inferences drawn from less theoretically motivated models—this in turn limits the level of detail and specificity gleaned for applied purposes. It has also been argued by others that inappropriate methods and analytic approaches can lead to misleading interpretations which can have detrimental effects on scientific literature as a whole (see Schmidt and Hunter 1996). Moreover, focusing exclusively on a global IQ score such as psychometric *g* can hide patterns of strengths and weaknesses which reduces transparency and could lead to inefficiencies in assessment and targeted intervention (see Kovacs and Conway 2019).

Additionally, an adverse impact is possible whereby a process is implemented where there are group differences in performance for a protected or vulnerable group of individuals. In the process of focusing solely on global IQ scores, such as in assessment methods implemented based on hierarchical models of CHC theory, there is still the possibility to create forms of adverse impact. Thus, we argue that any assessment of cognitive ability should investigate whether incorporating assessment design and practice based on formative models with POT would further reduce forms of adverse impact. Considering the diverse sets of needs of current students, we urge researchers to take care in specifying beyond the notion of general cognitive ability for applied purposes because it has been shown to be more biased and is ultimately less informative for how to best support students.

Collectively, we have presented empirical evidence from basic science and theory in cognitive psychology to demonstrate the utility of taking a modern perspective of intelligence with non-*g* POT focusing specifically on subprocesses of *g*. If we want to rise to the occasion by better supporting current students, then we argue that researchers and practitioners must take a more inclusive and less biased approach. We argue that more seriously considering a non-*g* POT perspective for future assessment research and practice offers a promising next step in that direction.

## 9. General Discussion

Spearman’s (1904) model and theory of intelligence were derived from European school children and ultimately became the justification for cognitive assessment practices and intelligence research (Gale Encyclopedia of Psychology 2013). A lot has changed since then; therefore, a theory and model of cognitive ability and intelligence from this particular sample may not fit well in today’s world. Considering where theories and models came from and how they can be improved to better align with students today is important for the design and development of cognitive assessments.

Historically, models of cognitive assessment came from correlational data. From this, we saw the development of a hierarchical common cause model of intelligence that led to innate views of intelligence (Galton 1869; Jensen 1998). These models were often applied and used to cause harm (see Russell 2009) and to inaccurately make causal claims about differences in cognitive ability and the ability to learn (see Strong 1913). This shows that we should take care in the development of our models and theories of intelligence, as well as the assumptions made, and the inferences drawn about ability. Taking into account current and projected demographic changes in the U.S., we should reconsider the role of cognitive assessment in educational equity by redefining what its role can be. The space of the educational landscape requires us to ensure that forms of assessment are fair today, and as outlined above they are not, so we must make changes.

We argue that the non-*g* model of POT can be used for equity and fairness. As a formative model non-*g* POT could help us reconsider the role of cognitive assessment, redefining what it is and how it works. This also helps us to begin to conduct research on how to build evidence to make better theories and test them through science. From our perspective, this can have the most benefit when we consider forms of intervention and differential responses to intervention. We argue that non-*g* POT theory and models can be used for these purposes because they can show us where to focus.

Based on POT, previous research has revealed that the most important cognitive processes are domain-general executive processes (Kovacs and Conway 2016; Shipstead et al. 2016; Kane et al. 2004; Engle et al. 1999) with domain-specific processes appearing to be secondary in importance (see Kovacs and Conway 2016, 2019). We also know that no cognitive ability task is process pure (Mashburn et al. 2021; Shipstead et al. 2016) and that each task involves some aspects of domain-general executive processes, and likely various domain-specific processes as well. Therefore, future work should focus on tackling diversity and inclusion concerns while prioritizing the most important cognitive processes involved in cognitive assessment tasks.

In terms of diversity and differences in cognitive processes, the forms of marginalization based on cultural or ethnic identity or neurodiversity can alter how individuals experience their environment and their developmental process (see PVEST model Spencer et al. 1997). Further, as the self develops in context so does cognition (see Prather 2021, also Spencer 1995), with much work supporting the idea that cognitive functions develop and are deployed differently based on forms of cultural and neurodiversity (Washington et al. 2018; Gutchess and Rajaram 2022; Thomas et al. 2023; Wang 2021; Ortiz and Oganes 2022). This emphasizes the importance of the context individuals navigate and what they experience in their developmental environments (Bronfenbrenner and Ceci 1994). These ideas align with more inclusive theories of intelligence such as Sternberg’s (1985) triarchic theory of intelligence and Gardner’s (1983) theory of multiple intelligences which both highlight the importance of cultural context for viewing intelligence but were limited by measurement issues for clinical applications. However, the non-*g* POT perspective is both amenable to diversity, equity, and inclusion concerns and is not limited by measurement issues in the way the more inclusive and culturally sensitive theories and models of intelligence are, as stated above.

Applying cognitive psychology research such as non-*g* POT to intelligence assessment is a good place to start for more equitable practices because non-*g* POT is an emergent model. It allows us to look at the level of specific subprocesses of *g* to see what is most important and for whom. Further, there is already preliminary empirical evidence supporting the utility and application of a non-*g* process overlap perspective of intelligence and ability. For example, some research on working memory training shows it is beneficial for various developmental and learning disabilities (Fuchs et al. 2020, 2022; Kofler et al. 2020; Zhang et al. 2018; Singh et al. 2022). Although some findings are mixed about the extent to which training working memory and domain-general executive processes is beneficial for far transfer (see Redick et al. 2013; Shipstead et al. 2012), the recent evidence outlined above for tailored intervention for more diverse needs is promising. There is also empirical evidence suggesting the importance of examining domain-general vs. more domain-specific differences for how to better tailor forms of intervention (Martin et al. 2020; Peng and Swanson 2022). More sophisticated modeling with network and advanced statistical methods also suggests that applying non-*g* POT-based models would be useful when thinking about which cognitive processes are most important for how to redesign intelligence models in subsequent research (McGrew et al. 2023). Therefore, non-*g* POT would provide a way to better understand cognition and diversity. This non-*g* POT approach can be adopted by both researchers and practitioners and, next, we detail recommendations for both.

### 9.1. Recommendations for Researchers

Several scholars have argued for research in cognitive psychology to be more inclusive culturally to improve our understanding of cognitive function and processes as well as the generalizability of empirical research (Gutchess and Rajaram 2022; Holden et al. 2022b; Prather 2021; Miller-Cotto et al. 2021; see also Roberts et al. 2020; Thomas 2020). These perspectives make the case that what people experience in their cultural context will influence their psychological orientations including their cognitive functions, their personal values, and their approaches to thinking and problem solving. For example, cultural differences have been shown to impact performance in cognitive tasks such as the Eriksen flanker task (Gutchess et al. 2021) which is known to involve *g* subprocesses related to executive control. Additional work has found cultural differences in perceptual and mnemonic performance (Gutchess and Sekuler 2019). As Kovacs et al. (2019) point out, there is a need for further investigation of inter- and intra-individual differences in the provision of subprocesses of *g* and WMC; this would also align with the idea that these provisions and mechanisms may differ from one person to the next; it is an empirical question the extent to which cultural and contextual factors influence these processes and their provisions to WMC as well.

Representation is another important consideration for research and for replicability purposes. The context in which research takes place and who is represented in that research matters for subsequent models and theory development. How people are included in research also matters, researchers need to be thoughtful in ensuring folks of diverse backgrounds are adequately represented and sampled. Society is ever evolving; both researchers and practitioners need to pay more attention to diversity, equity, inclusion, and fairness. Fairness should be taken into consideration in all aspects of science related to assessment design, implementation, and administration (AERA et al. 2014). All this is important when thinking about results and how to apply the research to best serve students across different backgrounds, identities, and needs.

In addition, science may not replicate or translate appropriately if samples are not diverse or adequately representative (Graves et al. 2021). This also limits for whom the different forms of intervention might be most effective (see Holden et al. 2022a, 2022b; Lindo 2006; Proctor et al. 2012). When we think about science at large, diversity and inclusion have an impact on replicability (see Feldon et al. 2023 for a recent example). Students today are increasingly diverse and often have multiple marginalized identities. This should also be kept in mind when we think about how to approach the work that will influence cognitive assessment theory and practice.

Future research work should adopt a non-*g* POT perspective, studying more important subprocesses of *g* and their roles in intelligence assessment. Researchers should examine differences in broad abilities across and within groups in terms of neurodiversity, linguistic diversity, and race/ethnicity. Intelligence assessment developers should be specifically concerned with ensuring that they can accurately and fairly assess the diverse students of today.

### 9.2. Recommendations for Practitioners

School neuropsychology is a field that focuses on understanding and assessing children’s processes of learning and academic development. As such, to achieve nondiscriminatory forms of assessment practice, we must consider that the brain’s organization and development are bound to the cultural context in which they unfold and seek to understand the impact of culture on language and neuropsychological performance (Ortiz and Oganes 2022). Inclusive forms of assessing student performance should recognize that cultural differences could impact a variety of cognitive processes including “decision speed, retrieval fluency, problem solving, auditory processing, acculturative knowledge acquisition, language proficiency, and other abilities” (Ortiz and Oganes 2022). In the future, school psychologists in both research and practice should focus more on examining the broad abilities and subprocesses of *g* from an equity perspective.

For practitioners to better align with recommendations regarding fair assessment practice, they must acknowledge and work to understand the ways that cultural and linguistic diversity corresponds with developmental differences. A step in the right direction would be working to increase diversity in the field of school psychology. A 2020 survey of the National Association of School Psychologists found that over 80% of the surveyed members identified as female, White, able-bodied, and monolingual (Goforth et al. 2021). Others have called for a need to recruit and retain more Black school psychologists as well (Proctor 2022; Blake et al. 2016). Given the shifting demographics in the country as a whole and within the student population, more diverse school psychologists would be beneficial to students for them to feel more comfortable in educational and assessment contexts and to see people who look like them in these positions. Increasing the diversity of practitioners helps to increase the number of people conducting research and working in practice who will understand and identify with forms of cultural and linguistic diversity which could have many positive impacts in assessment research and practice as well.

Another important step is for practitioners to consider using more inclusive assessments such as the KABC-II, DAS, and CAS. Though it should be noted that these assessments have some limitations as previously mentioned, they are the most equitable assessments available currently. Despite the fact that these were not the most used among practitioners, Aston and Brown (2021) found that a majority of school psychologists felt prepared to assess students from diverse backgrounds, but part of properly assessing these students is using fair assessments. Considering that the WISC is commonly used (Aston and Brown 2021; Sotelo-Dynega and Dixon 2014; Oakland et al. 2016), they and other test-makers should discuss and evaluate how their practices are inclusive and amenable to growing forms of diversity. Overall, developers of cognitive assessments should be updating their current practices to be fairer and more equitable for diverse groups. Part of this approach should include thoughtful examination of the theoretical bases of these assessments and shifting to non-*g* models of intelligence such as POT.

While more research is needed, practitioners must be aware of the fact that cognitive assessments have been found to be inequitable and should keep up to date with scientific studies examining how to make assessments more fair, equitable, and inclusive. The present work has highlighted the extent of under-representation of marginalized groups in not only the data collection of basic science on cognition and intelligence, but also in the sampling methods and standardization processes for many cognitive assessments, and in the intervention research.

## 10. Conclusions

At present, much more needs to be done to train practitioners in nondiscriminatory practices aiding them in differentiating psychological disorders and disabilities from differences originating from cultural differences (Ortiz and Oganes 2022). We must work to be more culturally sensitive in our approaches and non-*g* models can offer a first step in that direction. These non-*g* models might help us uncover how marginalized and racialized individuals could leverage and deploy their cognitive faculties in different ways and thus, such contextually and culturally adaptive responses should not be viewed as deficits but instead as diverse forms of functioning. Scientists and practitioners should work to rise to the occasion to better serve these populations. We have argued that assessments can be designed for everyone and that we do not need to get rid of them, but rather redesign them. Research and practice can be better designed by thinking about more inclusive, culturally sensitive, and representative research. By doing that, new, strong empirical evidence can be developed and then applied to practice. We argue that we start with a non-*g* approach with POT to re-envision and reconsider how we define, theorize about, model, conduct, and apply intelligence research to assessment design and practice.

## Data Availability

Not applicable.
